# A comprehensive review of post-harvest agricultural product deterioration signature volatile organic compounds

**DOI:** 10.1016/j.fochx.2025.102866

**Published:** 2025-08-04

**Authors:** Lu Sun, Junning Ma, Giorgia Purcaro, Gang Wang, Jing Jin, Fuguo Xing

**Affiliations:** a Institute of Food Science and Technology, Chinese Academy of Agricultural Sciences /Key Laboratory of Agro-Products Quality and Safety Control in Storage and Transport Process, Ministry of Agriculture and Rural Affairs, Beijing, 100193, PR China; b Gembloux Agro-Bio Tech, University of Liège, Passage des Déportés 2, 5030 Gembloux, Belgium

**Keywords:** Post-harvest, Agricultural products, Spoilage detection, Volatile organic compounds, Deterioration, Monitoring systems

## Abstract

Post-harvest management of agricultural products is crucial for minimizing spoilage and economic losses. Volatile organic compounds (VOCs) have emerged as effective indicators of early-stage deterioration, offering a promising approach to improving detection methods. This review examines the role of VOCs in spoilage identification, emphasizing key markers such as terpenes, ketones, esters, and aldehydes in fruits, vegetables, grains, and legumes. Various detection techniques—including spectrometry, electronic noses, spectroscopy, and sensor arrays—are evaluated and compared for their potential to assess spoilage and freshness by correlating their limits of detection (LOD) with typical VOC concentrations in agricultural scenarios. Future development trend in VOC research focus on enhancing sensor sensitivity, developing portable detection devices, integrating VOC monitoring with smart systems, and leveraging artificial intelligence for predictive analysis. These advancements aim to optimize post-harvest management strategies and improve food safety through more accurate and timely spoilage detection.

## Highlights

• A review and comparative analysis of food deterioration detecting techniques with a focus on VOCs.
• The signature VOCs of different food during deteriorations are reviewed.
• The development trends of the field and the innovative methods are discussed.

## 1 Introduction

Postharvest deterioration refers to the degradation in the quality of agricultural products after harvest, caused by factors such as improper handling, suboptimal storage conditions, and delays in processing (Nath et al., 2024). It results in quantitative losses due to a decrease in edible food mass, and qualitative losses related to reduced nutritional value, texture, color, and other sensory attributes, even though the food may still be technically edible. Preventing postharvest deterioration is essential for reducing postharvest losses (PHL) (Stathers et al., 2020). Effective management offers wide-ranging benefits, including optimizing food availability, reducing economic costs, conserving resources, preserving nutrients, preventing foodborne illness, extending shelf life, and minimizing waste. Adopting good postharvest practices is therefore key to minimizing food losses and ensuring the maximal utilization of agricultural products (Zhang, Zhang, et al., 2022).

Volatile organic compounds (VOCs) have emerged as powerful biomarkers for monitoring postharvest quality and detecting early signs of spoilage in agricultural commodities (Bonah et al., 2020; Giménez-Campillo et al., 2025). VOCs are low-molecular-weight compounds emitted as a result of metabolic processes in plant tissues and microorganisms. Their profiles change significantly in response to physiological stress, microbial activity, enzymatic degradation, and chemical oxidation—making them sensitive and non-invasive indicators of product freshness and integrity (Tiwari et al., 2020).

VOC-based sensing mechanisms primarily operate through chemical or physical transduction of VOC interactions into measurable electrical, optical, or spectral signals (Chen et al., 2022; Gu et al., 2020). In recent years, material innovation, system integration, and intelligent functionality are the keys to advancing the next generation of gas sensors toward high sensitivity, low power consumption, wearability, and smart applications (Zhu et al., 2022). Advances in nanomaterials and microfabrication have further enhanced the resolution, portability, and cost-efficiency of these detection platforms (Kwon et al., 2025). The rationale for employing VOCs as biomarkers lies in their early and often compound-specific emission patterns that precede visible spoilage. Unlike conventional microbiological or physicochemical tests, VOC-based methods offer rapid, real-time, and potentially non-destructive monitoring. In addition, the integration of VOC sensing technologies with smart agriculture frameworks has gained momentum. Innovations include the development of portable e-nose devices (da Silva et al., 2023), artificial intelligence(AI)-enhanced sensor arrays (Zhang et al., 2024), IoT-enabled monitoring platforms (Navarro et al., 2020), and machine learning algorithms capable of decoding complex VOC signatures (Ma et al., 2023) to detect contamination or classify spoilage stages. These multidisciplinary advances are moving the field toward real-time, on-site, and automated postharvest quality control systems.

**Fig. 1:**
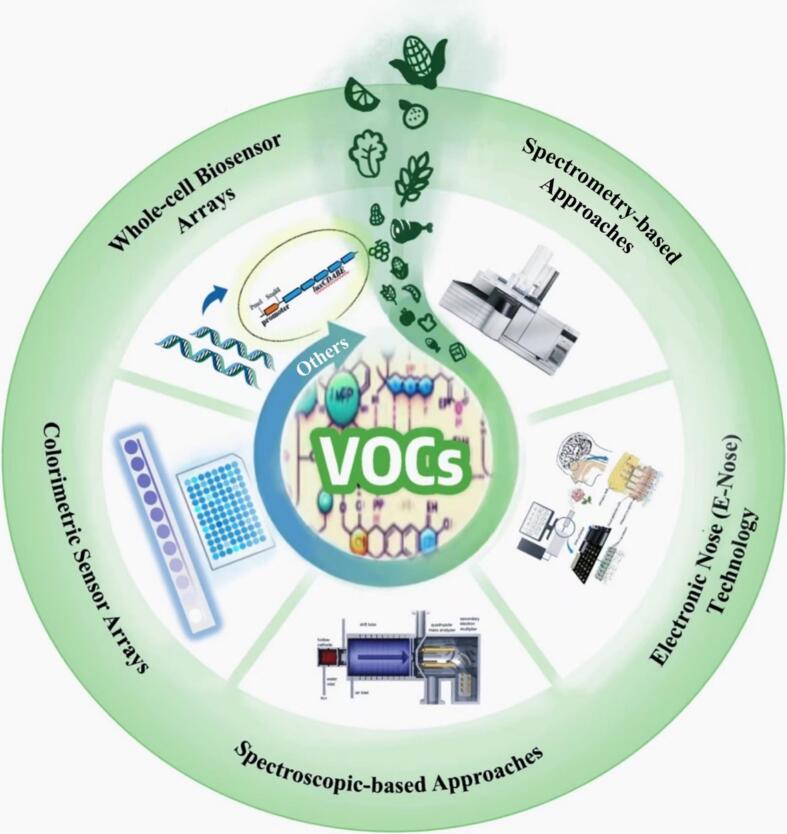
Scheme of post-harvest deterioration detection technique based on VOCs.

This review provides a comprehensive summary of VOC detection methods used to monitor postharvest deterioration of agricultural products over the past decades. It examines and evaluates various technologies, highlighting their advantages and limitations (Fig. 1). Additionally, it discusses the integration of VOC detection into smart systems for real-time surveillance and timely intervention. The review emphasizes the importance of addressing both economic and food safety risks posed by postharvest spoilage and outlines future research directions, including potential innovations and improvements in VOC-based detection strategies.

## 2 Volatile organic compounds (VOCs) in post-harvest deterioration

### 2.1 Major types and sources of VOCs in agricultural products

Agricultural products contain a wide variety of VOCs that play a significant role in shaping the aroma, flavor, and overall quality of these products. Consequently, they can serve as valuable physiological indicators for assessing the quality of agricultural products. Monitoring and evaluating the quality status of agricultural products can be achieved by analyzing the types and quantities of VOCs present. Common VOCs found in agricultural products include terpenes/terpenoids, ketones and aldehydes, ester compounds, alcohols, sulfurs, acids, nitrogen compounds, etc. As described in Table 1, Terpenes/terpenoids are the most abundant group of secondary plant metabolites mainly found in fruits, vegetables, and herbs, such as limonene in berries contributes to their fresh and zesty aromas (Gu et al., 2022). Linalool in basil gives them their unique herb scents (Walters et al., 2020). Ketones, aldehyde compounds, alcohol compounds, and ester compounds are common in fruits and grains, such as hexanal can contribute to the fresh aroma of strawberries, and ethyl acetate in apples contributes to the sweet aroma (Yang et al., 2021). Acid compounds such as acetic acid can be found in fruits and vegetables (Gu et al., 2022). Sulfur compounds like cysteine sulfoxides are prevalent in pungent vegetables such as garlic (Kovacevic et al., 2023). These VOCs can be considered as key biomarkers for monitoring the quality of agricultural products during storage. Detecting and analyzing these compounds allows for early intervention to prevent spoilage, thereby ensuring the quality and safety of food products (Tiwari et al., 2020). Notably, the complex aroma of an agricultural product is often the result of a diverse mixture of VOCs, influenced by a complex interplay of factors such as variety, ripeness, damage, storage conditions, and microbial contamination.

**Table 1:** The major type of VOCs in relation to the different agricultural products.

Types of VOCs	Products	Major biomarkers	References
Terpenes/Terpenoids	Fruit and vegetables	Limonen, Linalool	(Gu et al., 2022; Walters et al., 2020)
Ketones and Aldehydes	Grains and fruits	Hexanal	(Yang et al., 2021)
Ester Compounds	Grain, fruits, legumes	Ethyl Acetate	(Li et al., 2021; Wang et al., 2021)
Alcohols	Grains and fruits	3-Methyl-1-butanol, Ethanol	(Boriesson., 1999)
Sulfurs	Garlic	Cysteine sulfoxides	(Kovacevic et al., 2023)
Acids	Fruit, fermented foods	Acetic acid	(Gu et al., 2022)

VOCs in agricultural products can be classified into two subtypes based on their production and response to various factors: constitutive and induced. Constitutive VOCs are naturally and continuously produced while induced VOCs are generated as a response to a wide range of environmental stresses. Constitutive VOCs are naturally produced, and typically released with time. Yang et al. (2021) summarized the profile of VOCs change in apples that aldehyde was the main volatile component in the early stage, and then the alcohol content began to increase significantly, and the ester content increased with the decrease of aldehyde. Amundsen et al. (2023) observed that Lingonberries exhibited a significant decrease in the total concentration of phenolic compounds across five ripening stages. In contrast to constitutive VOCs, induced VOCs are produced in response to various abiotic and biotic stresses (Tiwari et al., 2020). Postharvest abiotic stress involves the temperature, relative humidity, oxygen levels, damage during transport, etc. Among the VOCs, terpenes, nitrogen-containing compounds, and phenolic compounds exhibit the most substantial susceptibility to abiotic stressors (Nagalingam et al., 2023). Moreover, when agricultural products are infected by bacteria and fungi, viruses, insects, or parasites, it can lead to biotic stress that generates VOCs as by-products of metabolism changes. These VOCs are closely linked to spoilage and fermentation, often resulting in noticeable changes to the flavor and aroma of the product.

### 2.2 Metabolic pathways leading to VOCs emission

As a consequence of agricultural products' interaction with biotic and abiotic factors, the VOCs are biosynthesized through interconnected pathways regulated by complex mechanisms. Recent advances in omics technology over the past years have led to a better understanding of plant VOCs biosynthesis (Picazo-Aragones et al., 2020). The biosynthesis of VOCs is closely linked to primary metabolism and the availability of energy sources. They can be roughly categorized into three classes based on their biosynthetic origin, including terpenoids, phenylpropanoids/benzenoids, fatty acid derivatives, and amino acid derivatives, along with some species- or genus-specific compounds that may not fit into these major classes. Dudareva et al. (2013) illustrate the interconnected biosynthetic pathways of VOCs with primary catabolic intermediates like acetyl-CoA, pyruvate, phosphoenolpyruvate (PEP), and erythrose-4-phosphate (E4P) as precursors (Fig. S1). C_18_ fatty acids, including linoleic acid and linolenic acid, are catalyzed by lipoxygenases and generate 9-hydroperoxy and 13-hydroperoxy derivatives of fatty acids. These intermediates turn into fatty acid derivatives, including methyl jasmonate and green leaf volatiles (Gu et al., 2022). The lipoxygenase pathway also can synthesize oxylipins, isoprene, carotenoid derivatives, indoles, phenolics, methyl salicylate, and aromatic VOCs. Sesquiterpenes, another group of VOCs, are synthesized through the cytosol mevalonate pathway (MVA), with acetyl-CoA as a key component, resulting in various intermediate metabolites (Dudareva et al., 2013). The methylerythritol phosphate (MEP) pathway occurs exclusively in plastids, and uses pyruvate as a precursor to create two common five‑carbon (C_5_) intermediates, isopentenyl diphosphate (IPP) and its allylic isomer, dimethylallyl diphosphate (DMAPP), leading to the synthesis of geranyl diphosphate (GPP) (Picazo-Aragones et al., 2020). In higher plants, the MVA and MEP pathways are interconnected through metabolic crosstalk (Bick & Lange, 2003). Geranyl diphosphate is converted to geranylgeranyl diphosphate (GGPP) with the help of the GGPase enzyme, ultimately producing volatile carotenoids. Geranylfarnesyl diphosphate (GFPP) is the recently discovered precursor of sesterterpenes (Sun et al., 2016). Monoterpenes, diterpenes, and hemiterpenes are also produced through the carotenoid biosynthesis intermediates. These 3-Deoxy-d-arabinoheptulosonate 7-phosphate (DAHP) pathway-based VOCs can be classified into different classes, including terpenoids, benzenoids, phenylpropanoids, derivatives of amino acids, and fatty acids. Among these classes, terpenoids are the largest followed by benzenoids and phenylpropanoids of aromatic origin (Tiwari et al., 2020). The advancements in the biosynthesis, ecological roles, and applications of plant VOCs have been summarized by Maffei et al. (2011).

While much attention has been given to the role of VOCs during plant growth, their role in post-harvest deterioration is now attracting increasing research interest. Since the volatilomics can characterize the physiological state of agricultural products before visible changes occur, it holds significant potential for early monitoring of deterioration, particularly in early mold detection (Jiang et al., 2023; Navarro et al., 2020).

## 3 Detection techniques for VOC-based spoilage detection

### 3.1 Spectrometry-based approaches

Spectrometry-based techniques are among the most widely used and valuable methods for establishing unique VOC profiles in food safety, fraud detection, freshness assessment, and quality evaluation (Chen et al., 2022). The identification of signature VOC compounds in deteriorated agricultural products for early pathogens monitoring based on spectrometry approaches is summarized in Table 2.

**Table 2:** Identification of signature VOCs emitted by deteriorated agricultural products for early pathogens monitoring based on spectrometry approaches^a^.

Foodstuff	Pathogens	Approaches	Signature VOC compounds	Pattern recognition	Approaches	References
Fragrant rice	*Aspergillus niger, Penicillium janthinellum, Penicillium alternatum, Penicillium funiculosum, Aspergillus flavus and Aspergillus parasitica*	GC-IMS	Alcohols, aldehydes, ketones, and esters	PCA	GC-IMS	(Chen et al., 2022)
Xiang Ling walnut	*Aspergillus flavus*	GC-IMS	Ethyl acetate-D, 3-methyl-2-butanol, and cyclohexanone	PCA	GC-IMS	(Wang et al., 2021)
Citrus (*Citrus reticulata* Blanco)	*Penicillium italicum*, *Penicillium digitatum* and *Lasiodiplodia theobromae*	GC–MS	dihy-drocarvone, *Z*-carveol, *cis*-beta-terpineol, *cis*-limonene 1,2-epoxide, D-carvone, 3-methyl-2-buten-1-ol, styrene and methyl (2-methyl-3- butene-2-yl) ether, and alpha-guajene	PCA and PLS-DA	GC–MS	(Wu et al., 2023)
Fuji apples	*Penicillium expansum*	GC–MS	(*E*)-hex-2-enal, 1-methoxy-3-methylbenzene, methyl heptanoate, diethyl carbonate, ethyl 2-phenylacetate, propyl octanoate, and ethyl decanoate	PCA	GC–MS	(Kim et al., 2018)
	*Botryosphaeria dothidea*		(E)-hex-3-enyl acetate, 1-methyl-4-propan-2-ylbenzene, 2-phenylethanol, α-terpinene, and α-terpinolene			
	*Alternaria alternata*		phenylmethanol, 2-ethylhexan-1-ol, and acetophenone			
Wheat Kernels	*Fusarium graminearum*	GC–MS	*F. graminearum* PH-1 infestation: 5-pentyl- cyclohexa-1,3-diene, 3-hexanone, and 1,3-octadiene. Zearalenone production:6-butyl-1,4-cycloheptadiene, hexahydro-3-methylenebenzofuran-2(3*H*)-one, and (*E*,*E*)-3,5-octadien-2-one	PCA and PLS-DA	GC–MS	(Ji et al., 2022)
Maize kernels	*Aspergillus flavus*	HS-GC-IMS	*Aspergillus flavus*: ethyl acetate-D and 3-hydroxybutan-2-one-D. AFB_1_ production: (E)-2-octenal-M, benzene acetaldehyde, (E)-hept-2-enal-M, 2-heptanone-D, and 2-pentyl furan	PCA	HS-GC-IMS	(Li et al., 2021)
Grape (Cabernet Sauvignon and Petit Manseng)	*Botrytis cinerea*, *Coniothyrium diplodilla (Speg.) Sacc*, *Colletotrichum* sp.	GC-IMS	Hexanal and (*E*)-2-hexenal	PCA, NAA	GC-IMS	(Li et al., 2022)
Strawberry	*Botrytis cinerea*	HS-MCC–IMS HS-SPME fast GC–MS	3-methylbutanal, cis-4-decenal, 2-methyl-1-butanol, 2-methyl-1-propanol, 1-octen-3-one and 1-octen-3-ol	Multivariate data analysis	HS-MCC–IMS HS-SPME fast GC–MS	(Vandendriessche et al., 2012)

a PCA, principal component analysis; PLS-DA, partial least squares discriminant analysis; NAA, nearest axis aligned; HS-MCC–IMS, headspace-multi-capillary column-ion mobility spectrometry; HS-SPME, headspace solid-phase microextraction.

Gas chromatography-mass spectrometry (GC–MS) has been widely applied in food volatilomics for the evaluation of food quality. GC–MS separates compounds in a sample based on their volatilities and interactions with the stationary phase of the GC column and identifies compounds based on the mass-to-charge ratio and their specific fragmentation (Wu, Cao, et al., 2023). It is particularly advantageous when analyzing complex mixtures and when the precise identification of individual components is essential. Its sensitivity generally allows for detection at the ppb level. However, the quantitative performance is highly dependent on the sample preparation strategy, some targets can be as low as ppt level after using solid-phase microextraction (SPME) and other enrichment methods. GC–MS has been successfully applied to the analysis of flavor profiles and spoilage markers in a wide variety of foods, including fruits, juices, meats, and cereals, with most target VOCs were present at concentrations above the ppb level (Kataoka et al., 2000; Baimatova & Gionfriddo, 2025). However, traditional GC–MS systems are typically bulky and are confined to laboratory settings, limiting their use for continuous and onsite monitoring in storage facilities or during shipment. Although advancements in device miniaturization have led to the development of portable GC–MS for onsite detection, its application in real-time monitoring is still lacking (Leary et al., 2023). Another mass spectrometry-based analytical technique proton transfer reaction mass spectrometry (PTR-MS) is often more compact and can be designed for portability, making them better suited for on-site and field applications, such as post-harvest storage air monitoring. PTR-MS is an online measurement based on the reaction of volatile molecules with H_3_O+ ions followed by subsequent detection of protonated molecules with a mass spectrometer (Weraduwage et al., 2022). For instance, research highlights that PTR-MS performs better in detecting real-time VOC changes like flavor release during ripening and distinguishing microbial volatile metabolites, with better sensitivity ranging from 0.1 to 0.5 ppb and lower detection limits, while GC–MS offers superior separation of closely related compounds (Mazzucotelli et al., 2022; Schuhfried et al., 2017). However, mass spectrometry-based approaches have their own drawbacks, including poor mass resolution, making it difficult to differentiate isobaric molecules, and a limited mass range for multiple ion monitoring. To address resolution challenges, GC–MS and PTR-MS coupled with a high-resolution mass analyzer has been introduced. An innovation, known as time-of-flight (TOF) mass analyzer, has emerged as a novel tool for plant metabolomics analysis due to its impressive high-throughput capabilities (Li et al., 2020). Other advanced mass analyzers, including Orbitrap and Fourier Transform Ion Cyclotron Resonance (FT-ICR), offer extremely high resolution, allowing them to be more precise in resolving very closely related ions, which has been already applied in profiling breathomics and soil volatilomics (Kuchikata et al., 2024; Malik et al., 2024). Therefore, adopting a collaborative approach that combines both methods for post-harvest agricultural product VOCs detection lays a solid groundwork for advancing monitoring and early warning technology in this field.

Here are some other notable hybrid techniques, including gas chromatography-ion mobility spectrometry (GC-IMS), selected ion flow tube-mass spectrometry (SIFT-MS), gas chromatography-electron capture detector (GC-ECD), gas chromatography-atomic emission detection (GC-AED), membrane introduction mass spectrometry (MIMS), etc. (Erler et al., 2020; Han & Lim, 2024). GC-IMS combines the separation power of gas chromatography with the rapid, high-sensitivity ion migration analysis of ion mobility spectrometry to identify and analyze chemical substances. The limits of detection (LOD) for GC-IMS are >0.1 ng g^−1^, which is generally lower in sensitivity compared to GC–MS. However, GC-IMS offers clear advantages in rapid screening and early-warning applications, particularly in agricultural settings where medium to high concentration composite gas pattern recognition is required. Therefore, it is more suitable for early warning food deterioration processes or scenarios requiring large-sample screening, such as sorting lines and storage monitoring. In recent year, it has been widely used for detecting volatiles in fruits and grains (Erler et al., 2020). For example, the typical concentration range of volatiles in pears is 0.1 to 20,000 ng g^−1^, including compounds such as (E)-2-hexen-1-ol, (E)-2-heptenal, benzaldehyde, 6-methyl-5-hepten-2-one, (E)-2-octenal, and acetophenone (Giménez-Campillo et al., 2025). GC-ECD detects molecules that can capture electrons, particularly monitor halogenated VOCs. It is widely applied in pesticide residue analysis, though it is less commonly used in quality monitoring (Han & Lim, 2024). MIMS is another direct MS analysis that requires VOCs to pass through a semi-permeable membrane into a mass spectrometer. This technique excels in continuous VOC monitoring and can be designed for portability (Richards et al., 2018). Emerging X-ray-based techniques are increasingly being investigated for structural analysis of VOCs, utilizing methods such as X-ray fluorescence (XRF) to detect atomic-level alterations linked to spoilage indicators. However, their broader application remains constrained due to high equipment costs and complex instrumentation requirements (Feng et al., 2021).

### 3.2 Electronic Nose (*E*-Nose) technology

With the development of electronic technology, an online detection method E-nose based on a semi-selective sensor array and pattern recognition is increasingly favored for rapid VOC detection (Gu et al., 2020). Among the most widely used are metal oxide semiconductor (MOS) sensors, which detect VOCs based on changes in electrical resistance upon gas adsorption onto a metal oxide surface. In addition to MOS, other types including metal oxide semiconductor field-effect transistors (MOSFET), piezoelectric crystal (PC), quartz crystal microbalance (QCM), electrochemical sensors, and surface acoustic wave (SAW) (Ali et al., 2023). MOSFETs offer enhanced sensitivity and rapid signal transduction by detecting gas interactions at the gate region of the transistor. PC and QCM sensors function by measuring frequency shifts resulting from the adsorption of VOCs onto the crystal surface, providing real-time detection (Fan et al., 2023). Electrochemical sensors operate based on redox reactions at an electrode and are particularly useful for detecting specific gas species with high selectivity. SAW sensors, which detect variations in acoustic wave propagation caused by gas adsorption, are noted for their excellent selectivity (Wu, Yuan, et al., 2023). Among all these sensor types, MOS sensors remain the most widely adopted in agricultural contexts due to their high sensitivity, low cost, and suitability for high-volume applications. Their LOD is typically ≥1 ppm, depending on the specific VOCs, usage scenarios, and sensor types. MOS sensors are sensitive to a wide range of agricultural VOCs and are particularly suitable for trend analysis of high-level or composite gases in greenhouse and storage monitoring. Other sensors, such as QCM and SAW, offer a comparable application range to MOS but, with the use of diversified coatings, can achieve broader VOC detection capabilities (Ali et al., 2023; Zytek et al., 2023).

As a representative example of an artificial olfactory system, *E*-nose is superior to many conventional methods by its noninvasive, fast, and user-friendly features, and has been applied to detect pathogen contamination in food in many recent works (Table 3). At present, commercially available E-nose uses pre-existing data (collected in previous experiments) and a trained model (from machine learning or statistical analysis) to recognize different VOC patterns, which can effectively be used to identify different VOC in food samples (Nouri et al., 2020). Some food contaminated by microorganisms metabolizes signature VOC compounds, which offer unique odor fingerprints that can be used for pathogen early identification and monitoring (Giungato et al., 2018; Gu et al., 2019). A comprehensive description of the application of E-nose for pathogen early monitoring has been thoroughly discussed by Bonah et al. (2020).

**Table 3:** Recent studies on food pathogens detection based on key VOCs components by Enose^b^.

Sample	Pathogen	Signature VOC compounds	Pattern recognition	References
Brown rice grain	*Aspergillus* sp.	Octane, 2,2,4,6,6-pentamethylheptane, decane, dodecane, toluene, ethanol, 1-pentanol, 1-hexanol, 1-octen-3-ol, 2-heptanone and 2-pentylfuran	PCA, LDA, SVM	(Jiarpinijnun et al., 2020)
Grains	*Aspergillus*	1-octanol and tetradecane	PCA	(Gu et al., 2019)
Apples	*Penicillium expansum*	Nitrogen oxides, Broad methane, Sulfur-containing organics, Aromatics, organic sulfides	PCA, PCA-DA, LDA, PLS-DA, KNN	(Guo et al., 2020)
Japonica rice (*Oryza sativa* subspecies. Japonica)	Fungal	1-octen-3-ol and 3-octanone	PCA	(Zhang, 2022)
Apple	*Staphylococcus. Salmonella, Shigella*	Acetone, ethanol, methanol, acetaldehyde, ammonia and propanol	PCA, HCA	(Ezhilan et al., 2018)
Peaches	*Botrytis cinerea*, *Monilinia fructicola* and *Rhizopus stolonifer*	Terpenes (e.g., β-myrcene and α-pinene) and aromatic compounds	PCA, PLSR	(Liu et al., 2018)
Peanut	*Aspergillus* sp. *(Aspergillus flavus, Aspergillus parastiticus,* and *Aspergillus ochraceus)*	Amines, ammonia, alcohols and ketones	PC- LDA, PLSR	(Shen et al., 2018)

b PCA, principal component analysis; LDA, linear discriminant analysis; SVM, support vector machine; PCA-DA, principal component analysis followed by discriminant analysis; HCA, hierarchical cluster analysis; PLS-DA, partial least squares discriminant analysis; KNN, K-nearest neighbor; PLSR, partial least squares regression.

Although E-nose allows for non-invasive detection, its application in agriculture is limited by challenges like sensitivity to temperature and humidity, difficulty in distinguishing volatile compounds and concentrations, and high costs for commercial use. Comprehensive reviews of the advantages and disadvantages of e-nose-based approaches have been discussed in detail in numerous research works (Ali et al., 2023; Shi et al., 2017).

### 3.3 Spectroscopic-based approaches

Spectroscopic techniques detect VOCs by examining the interactions between electromagnetic radiation and VOC molecules. These methods have garnered significant attention in food research due to their inherent advantages, including high biosafety, unique molecular fingerprinting capabilities, and strong penetration depth. This category encompasses a wide array of techniques such as infrared (IR) spectroscopy, Raman spectroscopy, near-infrared (NIR) spectroscopy, ultraviolet-visible (UV–Vis) spectroscopy, fourier transform infrared spectroscopy (FTIR), photoionization detection (PID), laser-induced fluorescence (LIF), cavity ring-down spectroscopy (CRDS), terahertz imaging, hyperspectral reflectance analysis, luminescence-based detection, and colorimetric imaging. Each method offers unique advantages: IR, Raman, and NIR spectroscopy provide molecular vibrational information that supports functional group identification, with IR and Raman offering more distinct fingerprinting capabilities, while NIR excels in rapid and non-destructive quantification (Niklas et al., 2021). In practical applications, Raman spectroscopy is more suitable for high-moisture samples (such as fruits and vegetables) due to its insensitivity to water. However, its key limitation is an extremely low scattering cross section, making trace gas detection with low LOD challenging. UV–Vis spectroscopy excels in analyzing conjugated compounds and pigments, suitable for rapid screening; FTIR offers fast, non-destructive analysis with high sensitivity, selectivity, and resolution, achieving detection limits as low as ppb levels. However, its high cost, susceptibility to matrix interference, and bulky equipment restrict its use in field applications (Zhou et al., 2022; Liu, Huo, et al., 2023; Epping & Koch, 2023). PID supports real-time monitoring and offers a strong balance between sensitivity and selectivity, especially effective for field applications (Rezende et al., 2019). CRDS achieves ultra-sensitive gas-phase measurements by extending optical path lengths; and hyperspectral imaging enables spatially resolved VOC detection in heterogeneous food matrices (Kwaśny & Bombalska, 2023). Terahertz imaging, though less mature, is gaining attention for its ability to penetrate packaging materials and detect early spoilage signals at the molecular level (Abina et al., 2023; Hindle et al., 2018).

Several hybrid sensing strategies have been developed by integrating complementary mechanisms. A notable example is the AI-enhanced ion mobility and mid-infrared spectroscopy (IMMS), which synergistically combines multi-dimensional signal features to achieve high-accuracy VOC identification (Zhu et al., 2023). Another emerging approach leverages guided-mode resonance (GMR) structures coupled with infrared spectroscopy to improve detection sensitivity (Tantiwanichapan et al., 2023). The GMR concept, applicable to both optical fiber and on-chip platforms, offers enhanced light–matter interaction. For instance, a graphene oxide-coated, nanopatterned fiber-tip GMR sensor has demonstrated selective and sensitive detection of ethylene and methanol, highlighting the potential of this integrated photonic design (Tabassum et al., 2017). Recent studies on agriculture product pathogens detection based on key VOCs by spectroscopic approaches were concluded in Table 4.

**Table 4:** Recent studies on food pathogens detection based on key VOCs components by spectroscopic approaches^c^.

Sample	Pathogen	Approaches	Signature VOC compounds	References
Peanuts	*Aspergillus* spp.	NIR	–	(Shen et al., 2018)
Crop	*–*	GMR	Ethylene and methanol	(Tabassum et al., 2017)
Agriculture products	–	FTIR	Acetone, ethanol, and isoprene	(Zhou et al., 2022)
Rice	*–*	LOPGP-FTIR	–	(Liu et al., 2023)
–	*–*	IMMS	Isopropyl alcohol	(Zhu et al., 2023)

c NIR, near Infrared; GMR, guided-mode resonance; FTIR, fourier transform infrared spectroscopy; LOPGP-FTIR, long optical path gas phase fourier transform infrared spectroscopy. IMMS, ion mobility and mid-infrared spectroscopy.

### 3.4 Other sensor arrays and nanotechnology-based approaches

Some other approaches rely on reactive interactions (chemical or biological) to detect VOCs. Similar to the *E*-nose, the colorimetric sensor is another main branch of olfactory visualization sensors. Colorimetric sensor arrays are optical arrays based on chemical-response colorants such as dyes and nano-porous pigments, depending on the color changes produced by the reaction between color-sensitive materials and VOCs, from where the odor information can be converted into digital images thus qualitatively or quantitatively analyzing the target attributes (Wang, Jiang, & Chen, 2021). The colorimetric sensor has been successfully applied to many fields of food and agriculture for many years, including food freshness, shelf-life monitoring, and quality analysis such as tea quality analysis (Li, Dong, et al., 2023), and food varieties distinction such as rice varieties discrimination (Arslan et al., 2022). The colorimetric sensor has recently been used in wheat mildew monitoring and has shown great potential as a noninvasive “odor visualization” monitoring technique (Li, Wang, et al., 2023). Previous studies have shown that the colorimetric sensor can maintain its stability and sensitivity at different ambient temperatures and humidity, which makes up for the defects of MOS-type E-nose (Arslan et al., 2022). However, different dyes often react to the interaction of the same VOCs in the mixture, resulting in the deficiency of the existing colorimetric sensor in quantifying VOCs (Duan et al., 2021). Many previous studies worked on the detection and monitoring of mildew wheat by the colorimetric-based sensor array (Lin et al., 2023; Wang, Mo, et al., 2021). Compared with the traditional detection methods, the colorimetric sensor makes it easier to achieve miniaturization and portability. At present, qualitative portable arrays have been tried to be used in food packaging and commercialized in the detection of food freshness. Guo, Guo, et al. (2020) combined cross-reactive colorimetric barcode combinatorics and deep convolutional neural networks to monitor meat freshness by the colorimetric portable sensor array. The portable prediction platforms can also be applied to detect metabolic VOCs of food spoilage. Drawing on its data processing tools, it can help the development of grain and oil mildew sensors.

The whole-cell biosensor based on synthetic biology is an emerging food safety rapid on-site detection technology. Whole-cell biosensors use live cells as sensing elements such as transcription factors and riboswitches and reporting components such as fluorescence and gases, etc. (Chen, Chen, Su, Guo and Liu, 2023). The sensing and reporting elements are coupled by gene expression regulation and coupling to form a simple gene circuit, converting information into a recognizable signal to detect the target substance. Compared with other early detection techniques, whole-cell biosensors exhibit strong anti-interference capability due to the relative stability of the living cell environment. At the same time, there is no need for complex sample pretreatment. The biosensor mass production speed is fast with a low manufacturing cost and is portable and user-friendly. These significant advantages give whole-cell biosensors great application potential and market value in field-operable real-time food early monitoring applications(Chalupowicz et al., 2020). However, their sensitivity is generally moderate, typically at the ppm level. Reports indicate that the lowest LOD for three infection-derived VOC markers—nonanal, 3-methyl-1-butanol, and 1-octen-3-ol—are 0.17 ppm, 2.03 ppm, and 2.09 ppm, respectively (Ma et al., 2020). As an onsite monitoring tool, whole-cell biosensors rely on biological activity and thus have relatively short shelf-life. Because of the risk of cell inactivation, it mostly serves research purposes yet and faces the problem of storage in practical applications in agriculture (Ma et al., 2023). Thus, sustained efforts are still needed to improve the capacity of whole-cell biosensors that are currently tested at the laboratory level.

Other innovative sensors include chemi resistors based on the change in electrical resistance, electrochemical sensors through electrochemical reactions, piezoelectric sensors based on quartz crystal vibration, conductometric sensors based on conducting polymers or nanomaterials, etc. (Ba Hashwan et al., 2023; Moon et al., 2022). They are often more suited for portable, real-time, and field applications, providing a versatile and cost-effective alternative for VOC detection. Recent studies on food pathogens detection based on key VOCs by relative interactions were concluded in Table 5.

**Table 5:** Recent studies on food pathogens detection based on other sensor arrays and nanotechnology-based approaches^d^.

Sample	Pathogen	Sensor type	Signature VOC compounds	References
Wheat	*Aspergillus flavus*	Colorimetric sensor	1-Octen-3-ol	(Duan et al., 2021; Lin et al., 2023; Wang et al., 2021)
Shelled peanuts and maize kernels	*Aspergillus flavus* strain ACCC 32656	Whole-cell biosensor	2-acetyltoluene, sulfurous acid, 2-ethylhexyl hexyl ester, ethyl propionate, 1-methyl-1H-pyrrole, 3,5-heptadiyn-2-one, and hexanal	(Ma et al., 2023)
Citrus fruit	*Penicillium digitatum*	Whole-cell biosensor	Limonene	(Chalupowicz et al., 2020)
Potato tubers	*Pectobacterium*	Whole-cell biosensor	1-octanol, phenylethyl alcohol, 2-ethyl hexanol, nonanal, and 1-octen-3-ol.	(Veltman et al., 2022)

d MOF, metal-organic framework.

To facilitate the selection of appropriate VOC detection techniques for various postharvest and food safety applications, it is essential to evaluate the strengths and limitations of each approach systematically. While each method—ranging from spectrometry-based systems and *E*-nose configurations to spectroscopic and emerging biosensor platforms—offers unique advantages in terms of sensitivity, specificity, or field deployability, they also come with trade-offs involving cost, scalability, and environmental robustness. Therefore, a comprehensive comparison of these methods with respect to critical performance indicators, including sensitivity, specificity, cost, scalability, application scope, and known limitations, is summarized in Table 6 to support technology matching in practical agricultural contexts.

**Table 6:** Comparison chart of main VOC detection techniques.

Technique		Limits of detection (LOD)⁎	Sensitivity	Specificity / Resolution	Cost	Scalability / Portability	Limitations	Applicability in agricultural scenarios	References
Spectrometry-based Approaches	**GC–MS**	ppt-ppb level (depend on sample preparation techniques)	High	High (*m*/*z*-based compound identification)	High	Low (Normally lab-based, portable device exist)	Bulky equipment, not suited for real-time or field use	Suitable for low concentrations of metabolites or disease signaling molecules in most agricultural products.	(Kataoka,et al., 2000; Baimatova & Gionfriddo, 2025)
	**PTR-MS**	0.1–0.5 ppb	Very High	Moderate	High	Moderate (Commercialized compact and semi-portable devices.)	Limited mass resolution, expensive, matrix interference	Suitable for on-site and field applications, such as post-harvest storage air monitoring.	(Mazzucotelli et al., 2022; Schuhfried et al., 2017).
	**GC-IMS**	0.1 ppb	High	Moderate	High	Low	High cost, low portability	Suitable for rapid screening and early warning	(Giménez-Campillo et al., 2025)
	**MIMS**	≥1 ppb	Moderate	Moderate	Moderate	Moderate	Membrane fouling, semi-selective, requires calibration	Suitable for realtime monitoring of VOC in the ambient air	(Ketola et al., 2002; (Richards et al., 2018).
	**X-ray-based techniques**	1–100 ppm	Low	Low	High	Low	High equipment costs, complex instrumentation requirements, not suitable for trace concentrations	Suitable for nondestructive detection of food, such as spoilage markers and high-concentration pesticide residue	(Feng et al., 2021).
Electronic Nose (E-Nose) Technology	**E-nose (MOS)**	≥1 ppm	Moderate to High	Moderate	Low	High	Cross-sensitivity to humidity/temperature, drift over time	Sensitive to diverse agricultural VOCs and suitable for trend analysis of high-level or composite gases in greenhouse and storage monitoring.	(Ali et al., 2023)
	**E-nose (SAW/QCM)**	≥1 ppm	Moderate to High	High	Moderate	High	Requires controlled conditions, limited robustness outdoors	Comparable to MOS in application range, but with diversified coatings for broader VOC detection.	
Spectroscopic-based Approaches	**IR/Raman/NIR Spectroscopy**	ppm level	Moderate to High	Moderate to High	Moderate to High	Moderate to High	Expensive, sensitive to matrix interference	Suitable for high-moisture samples (such as fruits and vegetables) Detecting trace VOCs is difficult.	(Niklas et al., 2021; Zhou et al., 2022; Liu et al., 2023; Epping & Koch, 2023)
	**UV–Vis Spectroscopy**	ppm level	Moderate	Moderate	Moderate	Moderate	Limited to chromophoric compounds, low spatial resolution	Suitable for common VOCs rapid screening.	
	**FTIR**	ppb level	Moderate to High	High	High	Moderate	Expensive, sensitive to matrix interference, limit application in the field	Infrared absorption is effective for crop metabolite detection but fails to detect homonuclear gases like N₂, O₂, and H₂. Limit field applicability.	
	**Photoionization detection**	ppb level (0.6 ppm for toluene)	High	Low to moderate	Low	High	Cannot distinguish individual VOC species without a pre-separation step (e.g., with GC).	Suitable for detecting phenolic or terpene VOCs.	(Rezende et al., 2019)
	**CRDS**	ppt level	Very High	High	High	Low	Complex setup, expensive optics, lab-restricted	Suitable for trace signal.	(Kwaśny & Bombalska, 2023).
	**Terahertz Imaging**	≥1 ppm	Moderate	Moderate	High	Low	High cost, limited availability, sensitive to matrix interference, immature technology	Suitable for agricultural product quality inspection, detecting volatile gases (such as H₂S, methanethiol, ethanol, ammonia, etc.) in packaged foods (e.g., salmon).	(Abina et al., 2023; Hindle et al., 2018).
	**Hybrid sensing strategies (IMMS)**	ppb level	High	High	High	Moderate	Cost significantly higher than traditional single sensor	Multi-mode joint detection is suitable for complex scenarios.	(Zhu et al., 2023)
Other Sensor Arrays and Nanotechnology-Based Approaches	**Colorimetric Sensor**	ppm level	Moderate	Moderate	Low	High	Semi-quantitative, dye cross-reactivity, limited specificity	Detects spoilage markers in agri-foods (e.g., wheat mildew), or enables real-time VOC monitoring in packaging for freshness assessment.	(Li et al., 2023; Liu et al., 2023)
	**Whole-cell Biosensor**	ppm level (nonanal, 3-methyl-1-butanol, and 1-octen-3-ol was 0.17-, 2.03-, and 2.09-ppm)	Moderate	High (target-dependent)	Low	High	Short shelf-life, cell stability issues, lab-stage maturity	Detect VOCs or pathogen metabolites released during infections to achieve non-invasive early diagnosis	(Ma et al., 2020； Veltman et al., 2022)

⁎ The scope of LOD depends on the specific VOC and usage scenarios. The data in the table is a typical range. Please refer to the reference for details.

## 4 Practical applications: VOC-based detection of agricultural product deterioration

### 4.1 Fruits and vegetables

Some studies have evaluated different novel VOC-based detection technologies, which can be effectively applied in the early monitoring of agricultural product deterioration. In studies on fruit and vegetable freshness, Voss et al. (2019) presented a promising prototype of an *E*-nose to perform the VOCs emitted in the peach growth cycle including the post-harvest stage, demonstrating a high accuracy for the reduced data set for 7 sensors. Zhang, Zhu, et al. (2022) conducted a correlation analysis of quality changes and shelf-life of postharvest apples based on E-nose and GC–MS, the prediction model offered an effective forecasting of apple shelf life at different temperatures. Ifmalinda (2022) showed the potential application of e-nose to identify mechanically damaged avocados. Similar applications have also shown great potential in fresh-cut vegetables, such as characterization spoilage markers in broccoli (Chen et al., 2019), green bell pepper (Chen et al., 2018), and iceberg lettuce (Ioannidis et al., 2018). In addition to predicting freshness and damage, VOC-based detection is also suitable for determining post-harvest diseases. Haghbin et al. (2022) assessed an experimental electronic nose system and machine learning for early detection and monitoring of *Botrytis cinerea* in Hayward kiwifruit based on odor-extracted information, demonstrating the effectiveness of the radial basis function neural network trained with CFS-selected features in achieving highly accurate classification. In previous research, E-nose has also been used in the detection and discrimination of common fungal pathogens in peaches (Liu et al., 2018), apples (Guo, Wang, et al., 2020), and pomegranates (Nouri et al., 2020). Fruits and vegetables generally have more prominent VOC markers, such as terpenes/terpenoids, ketones, esters, and alcohol (Gu et al., 2022; Walters et al., 2020). These VOC compounds are key contributors to fruits and vegetables' fruity and aromatic characteristics, while grains and legumes produce very few esters naturally, with their VOC profiles dominated by aldehydes, alcohols, and other compounds.

### 4.2 Grains and legumes

Considering the quality of grains and legumes in the post-harvest stage, sensors have been applied more to evaluate early mold contamination and predict the disease. Jiarpinijnun et al. (2020) visualized VOCs profiles of fungal infection during storage of Jasmine brown rice by E-nose coupled with chemometrics. Lin et al. (2019)presented a novel colorimetric sensor based on nanoscaled chemo dyes that can detect inert VOCs during the mildewing process of stored wheat. The infection of the wheat sample with *F. graminearum* also showed a specific relationship between the composition of fungal flora and other VOCs such as 5-pentyl-cyclohexa-1,3-diene, 3-hexanone, and 1,3-octadiene (Ji et al., 2022). Li et al. (2021) have shown that potential biomarkers specific to *A. flavus* contamination in maize kernels, and potential VOCs correlated with the level of mycotoxin AFB_1_. Grains and legumes typically have higher levels of aldehydes due to lipid oxidation, making aldehydes strong markers of rancidity and spoilage in these foods, such as hexanal in peanuts and maize (Ma et al., 2023). In addition, alcohol production is a major feature in grain spoilage, while only minimal alcohol formation during storage in legumes. Thus, alcohols are more likely to be found in grains as signature markers. Much previous research reported different colorimetric sensors to monitor key VOCs marker 1-octen-3-ol that is emitted after mold infection in wheat (Duan et al., 2021; Lin et al., 2023; Wang, Mo, et al., 2021).

## 5. Challenges and limitations

5

### 5.1 Complexity and diversity of Volatilomics

The differences in the types and concentrations of VOCs can vary widely based on factors like the source (e.g., sample variety), location (e.g., storage conditions, environmental factors), treatment (e.g., processing, handling, chemical treatments), microbial activity (e.g., bacteria and fungi), and time (e.g., aging, ripeness, and maturity). VOCs profiles vary among different types of agricultural products because of the different plant's metabolic processes, composition, and structural characteristics. In recent studies, approximately 360 VOCs have been identified for strawberry aromas, and 200 VOCs have been identified in raspberries (Song et al., 2016). This complexity makes it difficult to detect, identify, and quantify all relevant VOCs accurately, especially when they are present at trace levels. Analyzing this variability is crucial for quality control, food safety, and flavor and aroma assessments. It can also help identify factors that affect the shelf life and overall quality of these products.

### 5.2 Interference from external factors

The interference of external factors in detection technologies is a critical challenge in terms of the variety of VOCs composition. The first is the interference of external odors. Since detecting deterioration through VOCs in agricultural products is an indirect method, it requires sampling and analyzing the VOCs emitted by the sample. This process can be easily influenced by other VOCs present in the environment. Such as residues of pesticides and fertilizers, and interfering gas in the air may be mixed with target VOCs, leading to misleading detection results. For example, high temperatures may increase volatile release, changes in humidity may affect the response of sensors, and atmosphere control may influence plant metabolism and microorganism VOCs emissions (Cellini et al., 2021). Especially in open agricultural settings, changes in meteorological conditions can impact the diffusion and concentration of volatile compounds in the storage. The composition of VOCs in agricultural products is also highly influenced by environmental stresses, which produce induced VOCs in response to various biotic and abiotic stresses. As mentioned above, the stressors mainly include microorganism infection, pest attacks, temperature and humidity variations, and other environmental stressors (Tiwari et al., 2020). These may lead to changes in the type and concentration of VOCs produced by agricultural products, thus interfering with the detection results (Murali-Baskaran et al., 2022).

### 5.3 Standardization and validation of detection methods

Standardization and validation are crucial for ensuring accuracy and reproducibility in volatilomics. However, the lack of standardized protocols for VOC sample collection, processing, and analysis leads to inconsistent results across studies, affecting reliability. Different extraction methods, like GC–MS for specific components and the electronic nose for overall patterns, can produce varying VOC profiles. For instance, none of the hundreds of identified VOCs in strawberries were consistently reported across studies (Ulrich et al., 2018). To improve consistency, combining multiple technologies and establishing standard samples and procedures is recommended. Validation involves assessing calibration, accuracy, precision, sensitivity, and stability to ensure reliable results. Calibration curves and field tests help correct for instrument drift and environmental interference. In field conditions, environmental factors like temperature, humidity, and airflow can affect the instrument's readings. Calibration in these conditions ensures that measurements are accurate in the real world. The field test can help researchers correct errors caused by instrument drift, external factor interference, or sensitivity changes (Hong et al., 2023).

Overall, to address the challenge of accurate, reliable, and reproducible VOC detection in field sampling, it is essential to regularly calibrate instruments using reference standards and perform multiple sampling trials to ensure reproducibility. Standardizing VOC extraction and collection methods can minimize variability, while comparing results with laboratory standards helps verify accuracy. Sensitivity should be assessed by testing detection limits and accounting for environmental background noise. Controlling environmental factors, using field blanks, and cross-validating with multiple detection technologies can further enhance reliability. Monitoring and correcting instrument drift, performing on-site field tests, and implementing standardized reporting protocols are also critical to improving data consistency and comparability across studies.

## 6 Integration into smart monitoring systems

### 6.1 Internet of things (IoT) applications in post-harvest monitoring

IoT technologies and intelligent sensing involve connecting devices through networks to achieve interconnectivity and intelligent control (Fig. S2). These devices are usually equipped with sensors, software, and other technologies to collect and exchange data, thereby improving efficiency and accuracy by data aggregation, real-Time feedback, redundancy identification, and remote updates (Lutz & Coradi, 2022). At present, there have been a large number of applications of the IoT in agriculture, such as in the aspect of smart farming, precision agriculture, resource management, soil management, pest and disease monitoring, etc. (Navarro et al., 2020). In addition to monitoring agriculture products in the field, they are becoming an indispensable practice of post-harvest agricultural monitoring, such as environment management that can remotely control the storage environments, disease prediction that can early monitor pest or microorganism contamination. Sanjeevi et al. (2020) introduced a post-harvest hierarchical model based on ontology, facilitating the IoT-driven prevention of post-harvest losses and the accurate differentiation of healthy sekai-ichi apples. Similarly, a real-time IoT-based monitoring system continuously tracks temperature, humidity, luminosity, and gas concentrations in cold storage, automatically alerting personnel when conditions exceed safe thresholds(Afreen & Bajwa, 2021). The IoT-based notification system can integrate cloud data to enable unified management of farms and granaries with similar storage environments within the same region. When IoT sensors detect signs of spoilage in any granary, real-time data, such as temperature fluctuations or volatile organic compound levels, are uploaded to the cloud and used to alert other granaries with similar environmental conditions. This allows for quicker responses and prevention of environmental changes or anomalies, thereby enhancing overall management efficiency and ensuring food security.

Smart monitoring systems leverage agricultural data to build AI models, with real-time data collection and automation key to advancing the industry. These systems offer benefits in post-harvest agriculture, such as disease detection and efficient data analysis. However, challenges such as external interference affecting data accuracy and increased risks from technological complexity, privacy, and security require robust intrusion detection systems and IoT-specific architectures to effectively mitigate these security risks (Qaddos et al., 2024).

### 6.2 Artificial intelligence (AI) and machine learning for data analysis

Artificial intelligence, particularly machine learning, has become indispensable in transforming raw sensor data into actionable insights for post-harvest quality control. AI algorithms enable the system to automatically learn from multidimensional environmental and volatile compound data, allowing for classification, forecasting, and pattern recognition without the need for manual rule-based programming (Lutz & Coradi, 2022). Pre-processing techniques—such as baseline correction, normalization, and data compression—are typically employed to reduce noise and standardize sensor responses, enhancing the reliability of subsequent analysis (Andre et al., 2022).

Typically, VOC information is processed using two primary categories of algorithms: statistical models and intelligent model analysis (Kim et al., 2022). Statistical methods, including principal component analysis (PCA), linear discriminant analysis (LDA), and support vector machines (SVM), are commonly applied for dimensionality reduction and visualization of sensor response patterns. These methods are computationally efficient, highly interpretable, and particularly effective for small datasets where linear relationships exist between features and classes. They are commonly used for exploratory data analysis, visual separation of classes, and quality classification under well-controlled experimental conditions. However, their capacity to model nonlinear interactions is limited, and their performance may degrade in the presence of noise or complex feature structures (Moshayedi et al., 2023). In contrast, intelligent methods such as artificial neural networks (ANN), multilayer perceptron (MLP), k-nearest neighbors (kNN), and tree-based methods like decision tree (DT), random forest (RF) and XGBoost offer powerful alternatives. These intelligent algorithms are particularly advantageous when dealing with large-scale, high-dimensional datasets or in dynamic sensing environments where adaptability and high prediction accuracy are required (Kim et al., 2022; Wang et al., 2022). Nevertheless, these algorithms are often criticized for their black-box nature, their susceptibility to overfitting when training data is limited (Chowdhury et al., 2022; Christmann et al., 2022). In real-time applications, latency and computational overhead present further constraints, requiring lightweight or optimized architectures to support responsive deployment in embedded systems (Li et al., 2023; Assimakopoulos et al., 2024). Advanced models designed to address challenges involve deep learning architectures such as convolutional neural networks (CNNs) and long short-term memory (LSTM), explainable AI (XAI), as well as evolutionary approaches like AdaBoost and genetic algorithms, all of which offer enhanced capability in handling complex, nonlinear, and high-dimensional odor datasets (Natarajan et al., 2024; Wang & Liu, 2024).

Algorithm selection should be guided by data scale, task complexity, interpretability, and available resources. Statistical models (e.g., PCA, LDA, SVM) are suitable for small, interpretable tasks, while intelligent models (e.g., RF, XGBoost, CNNs) perform better in high-dimensional, real-time settings. Hybrid approaches combining both are increasingly favored for balancing accuracy and transparency. For applications demanding traceability, integrating XAI is recommended. Additionally, auto-machine learning offers a promising solution to automate model selection and optimization, lowering deployment barriers. In practice, different machine learning models have proven particularly effective in tasks such as AI-enhanced mid-infrared gas spectroscopy to track Isopropyl alcohol biomarker (Zhu et al., 2023), detecting fungal infections like gray mold in stored kiwifruit (Haghbin et al., 2022), and classifying produce based on ripeness or microbial spoilage VOCs (Ma et al., 2023; Voss et al., 2019). Furthermore, feature extraction algorithms can automatically identify the most relevant volatile signatures from massive datasets, and multimodal fusion techniques integrate data from multiple sensing platforms can improve predictive accuracy (Lee, 2023). Future research should emphasize interpretable, resource-efficient AI architectures that can adapt to dynamic environmental conditions while ensuring scalability, traceability, and robustness in real-world deployments.

### 6.3 Real-time monitoring and intelligent decision support

Building on the synergy between IoT infrastructure and AI algorithms, real-time monitoring systems are capable of dynamically evaluating the freshness and safety of stored agricultural products. These systems utilize VOC fingerprint sensing and environmental data streams—such as temperature, humidity, and gas composition—which are uploaded to cloud servers and interpreted by trained AI models. The resulting outputs inform intelligent alerts, actionable recommendations, or automatic control responses aimed at preserving quality and reducing spoilage losses (Qaddos et al., 2024). For example, the integration of AI and IoT has enabled the development of systems that predict AFB_1_ contamination based on environmental trends (Moshayedi et al., 2023) and others that classify grain quality or monitor oxidative stress responses in crops (Ebrahimi et al., 2014; Lew et al., 2020). The continuous tracking of VOCs and instant feedback to users allows for a proactive rather than reactive approach to post-harvest management.

As these smart systems evolve, they are expected to not only support food supply chain resilience but also drive sustainability, cost-efficiency, and eco-friendly through precise environmental control and waste reduction. Future development will likely focus on adaptive AI models, secure data architectures, and low-power edge computing to further enhance responsiveness, scalability, and integration.

## 7 Development trends and future prospects

### 7.1 Current development trends of detection technologies

The current technological landscape of VOCs detection mainly includes a combination of conventional detection technologies with miniaturization technology. Many sensor technologies have been applied to VOCs detection in agricultural product deterioration, such as electronic noses and gas sensors. There are also many notable recent advancements based on VOCs detection such as IoT Integration, sensor miniaturization, microfabrication and nanotechnology, self-powered sensing systems, AI-enabled real-time monitoring, selective sensors, microelectromechanical systems, printed and flexible electronics technologies, microfluidic systems, smartphone-based gas detection, and wireless communication (Epping & Koch, 2023). Advances in these systems continue to drive innovation in the field of VOCs detection, thereby the device is becoming more compact, intelligent, and portable while maintaining high analytical performance.

The main challenges of these advances lie in the manufacture of appropriate electronic components (acquisition and transmission modules), signal processing, and software for data collection, such as all major components of the GC system have been reduced. In these cases, instrument sensitivity can be adjusted by modifying the input flow rate. Conducting environmental air analysis or headspace sampling in odorless bags or gas canisters is the simplest option, minimizing the possibility of obtaining artificial results. However, when these options are impractical (e.g., when gas samples are too small or the content of target compounds is trace), the use of VOCs adsorbent materials to concentrate VOCs samples can be considered (Cellini et al., 2021). You et al. (2020) reported a portable gas chromatograph featuring a carbon nanotube sponge preconcentrator, offering improved sensitivity and detection limits for trace VOCs in air samples. Dey et al. (2020) introduced a NiO/ZnO-based p–n junction single-diode device capable of selectively sensing multiple VOCs simultaneously by adjusting the bias voltage. Huang et al. (2021) developed a battery-powered portable *E*-nose system using multiple metal oxide gas sensors and machine learning algorithms to detect and classify VOCs in wine. While most current commercial E-nose devices rely on MOS-type gas sensor arrays, ongoing research is exploring alternative materials, such as two-dimensional transition metal carbides/nitrides (MXenes), which are gaining attention for improving gas sensing sensitivity (Chen et al., 2020). The development of VOCs collection and device miniaturization has greatly advanced VOCs measurements, proving particularly useful for indoor storage assessments and field studies.

### 7.2 Development of portable and on-site detection systems

Portable and on-site detection enables real-time analysis directly at the point of storage or processing of agricultural products, eliminating delays associated with transporting samples to centralized laboratories. Recent advancements have demonstrated the potential for visualizing VOC fingerprint differentials using portable platforms, such as two-dimensional code-configured E-nose systems. For example, Conrado et al. (2021) developed a simple, non-invasive, paper-based optoelectronic nose arranged in a QR code format to assess olive oil aroma. Similarly, Chang et al. (2018) introduced a photonic crystal-based sensor fabricated via a “nanoscale easy tear (NET)” process, inspired by the concept of tearable packaging. This compact, low-cost sensor enables effective on-site monitoring through colorimetric responses.

Integration with smartphones further enhances the usability of these systems by leveraging existing hardware for data visualization, user interface, and wireless communication. Li et al. (2019) developed a smartphone-based platform for plant disease diagnosis using a disposable colorimetric sensor array capable of detecting leaf volatiles at ppm levels within one minute. Lew et al. (2020) presented a Raspberry Pi system with a charge-coupled device camera that replicates smartphone imaging functionality for real-time and low-cost VOC monitoring.

### 7.3 Development of multi-parameter monitoring

**Fig. 2:**
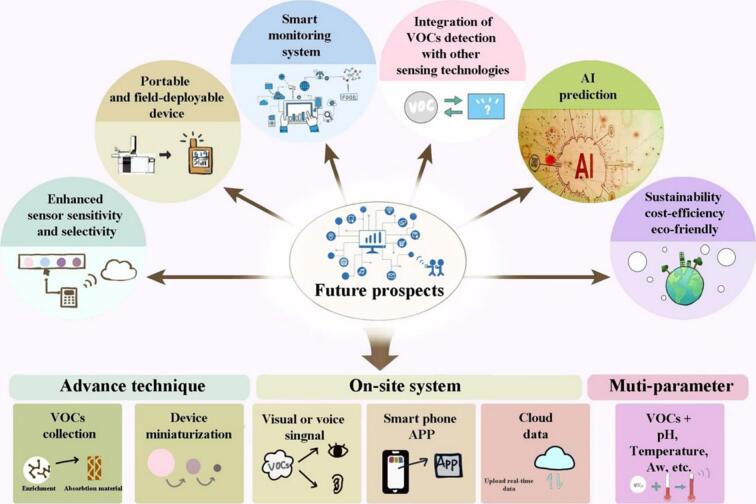
Future prospects and research directions of post-harvest agricultural product deterioration detection based on VOCs.

Spoilage is a complex process influenced by various factors, and a comprehensive assessment requires consideration of multiple parameters simultaneously (Snyder et al., 2024). In addition to VOCs analysis, assessing different odor characteristics, monitoring the pH and acidity of agricultural products, conducting microbiological and texture analyses, temperature and humidity monitoring, conductivity measurement, water activity determination, etc., are also crucial for understanding chemical changes and the growth of spoilage microorganisms in agricultural products (Fan et al., 2023). A comprehensive assessment enables a more nuanced understanding of the factors influencing product quality and facilitates timely interventions to mitigate spoilage risks (Fig. 2).

## 8 Conclusion

VOCs represent sensitive and dynamic chemical markers for the early detection of spoilage and quality degradation in post-harvest agricultural products. This review summarizes key VOC signatures associated with deterioration and evaluates recent advances in detection technologies, including spectrometry, spectroscopic methods, electronic noses, and sensor arrays. We present a comparative evaluation of their performance, LOD, applicability, and constraints in agricultural scenarios, as well as emerging trends, highlighting their potential to enhance real-time decision-making in storage, transportation, and shelf-life management. While early VOC detection holds clear promise for reducing food loss and safeguarding consumer health, several challenges remain. These include the diverse and variable metabolic profiles of VOCs, susceptibility to environmental interference, and the lack of standardized protocols for method validation. Furthermore, high instrumentation costs continue to hinder large-scale deployment, highlighting the need for scalable, cost-effective solutions to facilitate broader adoption in the field.

## CRediT authorship contribution statement

**Lu Sun:** Writing – original draft, Visualization, Investigation, Formal analysis. **Junning Ma:** Investigation, Formal analysis. **Giorgia Purcaro:** Writing – review & editing, Validation, Formal analysis. **Gang Wang:** Validation. **Jing Jin:** Writing – review & editing, Supervision. **Fuguo Xing:** Writing – review & editing, Supervision, Funding acquisition.

## Declaration of competing interest

The authors declare that they have no known competing financial interests or personal relationships that could have appeared to influence the work reported in this paper.

## Data Availability

The authors do not have permission to share data.

## Appendix A Supplementary data

Supplementary material Image 1

## References

[bb0005] Abina A., Puc U., Jazbinšek M., Zidanšek A. (2023). Analytical gas sensing in the terahertz spectral range. Micromachines.

[bb0010] Afreen H., Bajwa I.S. (2021). An IoT-based real-time intelligent monitoring and notification system of cold storage. IEEE Access.

[bb0015] Ali A., Mansol A.S., Khan A.A., Muthoosamy K., Siddiqui Y. (2023). Electronic nose as a tool for early detection of diseases and quality monitoring in fresh postharvest produce: A comprehensive review. Comprehensive Reviews in Food Science and Food Safety.

[bb0020] Amundsen M., Hykkerud A.L., Kelanne N., Tuominen S., Schmidt G., Laaksonen O., Aaby K. (2023). Composition of sugars, organic acids, phenolic compounds, and volatile organic compounds in lingonberries (Vaccinium vitis-idaea L.) at five ripening stages. Foods.

[bb0025] Andre R.S., Mercante L.A., Facure M.H.M., Sanfelice R.C., Fugikawa-Santos L., Swager T.M., Correa D.S. (2022). Recent Progress in amine gas sensors for food quality monitoring: Novel architectures for sensing materials and systems. ACS Sensors.

[bb0030] Arslan M., Zareef M., Tahir H.E., Guo Z., Rakha A., Xuetao H., Khan M.R. (2022). Discrimination of rice varieties using smartphone-based colorimetric sensor arrays and gas chromatography techniques. Food Chemistry.

[bb0035] Assimakopoulos F., Vassilakis C., Margaris D., Kotis K., Spiliotopoulos D. (2024). Artificial intelligence tools for the agriculture value chain: Status and prospects. Electronics.

[bb0040] Ba Hashwan S.S., Khir M.H.M., Nawi I.M., Ahmad M.R., Hanif M., Zahoor F., Junaid M. (2023). A review of piezoelectric MEMS sensors and actuators for gas detection application. Discover nano.

[bb0045] Baimatova N., Gionfriddo E. (2025). Needle trap devices in analytical chemistry: A critical review of development, applications, and future perspectives. Analytical Chemistry.

[bb0050] Bick J.A., Lange B.M. (2003). Metabolic cross talk between cytosolic and plastidial pathways of isoprenoid biosynthesis: Unidirectional transport of intermediates across the chloroplast envelope membrane. Archives of Biochemistry and Biophysics.

[bb0055] Bonah E., Huang X., Aheto J.H., Osae R. (2020). Application of electronic nose as a non-invasive technique for odor fingerprinting and detection of bacterial foodborne pathogens: A review. Journal of Food Science and Technology.

[bb0060] Cellini A., Spinelli F., Donati I., Ryu C.M., Kloepper J.W. (2021). Bacterial volatile compound-based tools for crop management and quality. Trends in Plant Science.

[bb0065] Chalupowicz D., Veltman B., Droby S., Eltzov E. (2020). Evaluating the use of biosensors for monitoring of Penicillium digitatum infection in citrus fruit. Sensors and Actuators B: Chemical.

[bb0070] Chang H.K., Chang G.T., Thokchom A.K., Kim T., Park J. (2018). Ultra-fast responsive colloidal-polymer composite-based volatile organic compounds (VOC) sensor using nanoscale easy tear process. Scientific Reports.

[bb0075] Chen H.-Z., Zhang M., Bhandari B., Guo Z. (2018). Evaluation of the freshness of fresh-cut green bell pepper (Capsicum annuum var. grossum) using electronic nose. Lwt.

[bb0080] Chen H.-Z., Zhang M., Guo Z. (2019). Discrimination of fresh-cut broccoli freshness by volatiles using electronic nose and gas chromatography-mass spectrometry. Postharvest Biology and Technology.

[bb0090] Chen S., Chen X., Su H., Guo M., Liu H. (2023). Advances in synthetic-biology-based whole-cell biosensors: Principles, genetic modules, and applications in food safety. International Journal of Molecular Sciences.

[bb0095] Chen T., Chen X., Meng L., Wei Z., Chen B., Wang Y., Chen H., Cheng Q. (2022). Characteristic fingerprint analysis of the moldy odor in Guangxi fragrant rice by gas chromatography - ion mobility spectrometry (GC-IMS). Analytical Letters.

[bb0100] Chen W.Y., Jiang X., Lai S.N., Peroulis D., Stanciu L. (2020). Nanohybrids of a MXene and transition metal dichalcogenide for selective detection of volatile organic compounds. Nature Communications.

[bb0105] Chowdhury M.A.Z., Rice T.E., Oehlschlaeger M.A. (2022). VOC-net: A deep learning model for the automated classification of rotational THz spectra of volatile organic compounds. Applied Sciences.

[bb0110] Christmann J., Rohn S., Weller P. (2022). Finding features - variable extraction strategies for dimensionality reduction and marker compounds identification in GC-IMS data. Food Research International.

[bb0115] Conrado M., Sequinel J.A., Dias R., Silvestre B.C., Batista M.A.D., Petruci J.F.D.S. (2021). Chemical QR code: A simple and disposable paper-based optoelectronic nose for the identification of olive oil odor. Food Chemistry.

[bb0120] Dey S., Nag S., Santra S., Ray S.K., Guha P.K. (2020). Voltage-controlled NiO/ZnO p-n heterojunction diode: A new approach towards selective VOC sensing. Microsystems & Nanoengineering.

[bb0125] Duan Y., Lin H., He P., Chen Q. (2021). Detection of volatile marker in the wheat infected with aspergillus flavus by porous silica nanospheres doped Bodipy dyes. Sensors and Actuators B: Chemical.

[bb0130] Dudareva N., Klempien A., Muhlemann J.K., Kaplan I. (2013). Biosynthesis, function and metabolic engineering of plant volatile organic compounds. New Phytologist.

[bb0135] Ebrahimi E., Mollazade K., Babaei S. (2014). Toward an automatic wheat purity measuring device: A machine vision-based neural networks-assisted imperialist competitive algorithm approach. Measurement.

[bb0140] Epping R., Koch M. (2023). On-site detection of volatile organic compounds (VOCs). Molecules.

[bb0145] Erler A., Riebe D., Beitz T., Lohmannsroben H.G., Grothusheitkamp D., Kunz T., Methner F.J. (2020). Characterization of volatile metabolites formed by molds on barley by mass and ion mobility spectrometry. Journal of Mass Spectrometry.

[bb0150] Ezhilan M., Nesakumar N., Jayanth Babu K., Srinandan C.S., Rayappan J.B.B. (2018). An electronic Nose for Royal Delicious Apple Quality Assessment - A tri-layer approach. Food Research International.

[bb0155] Fan M., Rakotondrabe T.F., Chen G., Guo M. (2023). Advances in microbial analysis: Based on volatile organic compounds of microorganisms in food. Food Chemistry.

[bb0160] Feng X., Zhang H., Yu P. (2021). X-ray fluorescence application in food, feed, and agricultural science: a critical review. Critical Reviews in Food Science and Nutrition.

[bb0165] Giménez-Campillo C., Pastor-Belda M., Campillo N., Arroyo-Manzanares N., Viñas P. (2025). Fingerprinting of volatile organic compounds and discrimination of pear samples by gas chromatography-ion mobility spectrometry. Food chemistry.

[bb0170] Giungato P., Di Gilio A., Palmisani J., Marzocca A., Mazzone A., Brattoli M., Giua R., de Gennaro G. (2018). Synergistic approaches for odor active compounds monitoring and identification: State of the art, integration, limits and potentialities of analytical and sensorial techniques. TrAC Trends in Analytical Chemistry.

[bb0175] Gu I., Howard L., Lee S.-O. (2022). Volatiles in berries: Biosynthesis, composition, bioavailability, and health benefits. Applied Sciences.

[bb0180] Gu S., Chen W., Wang Z., Wang J., Huo Y. (2020). Rapid detection of aspergillus spp. infection levels on milled rice by headspace-gas chromatography ion-mobility spectrometry (HS-GC-IMS) and E-nose. Lwt.

[bb0185] Gu S., Wang J., Wang Y. (2019). Early discrimination and growth tracking of aspergillus spp. contamination in rice kernels using electronic nose. Food Chemistry.

[bb0190] Guo L., Wang T., Wu Z., Wang J., Wang M., Cui Z., Ji S., Cai J., Xu C., Chen X. (2020). Portable food-freshness prediction platform based on colorimetric barcode combinatorics and deep convolutional neural networks. Advanced Materials.

[bb0195] Guo Z., Guo C., Chen Q., Ouyang Q., Shi J., El-Seedi H.R., Zou X. (2020). Classification for penicillium expansum spoilage and defect in apples by electronic nose combined with chemometrics. Sensors.

[bb0200] Haghbin N., Bakhshipour A., Mousanejad S., Zareiforoush H. (2022). Monitoring Botrytis cinerea infection in kiwifruit using electronic Nose and machine learning techniques. Food and Bioprocess Technology.

[bb0205] Han N.S., Lim J.S. (2024). Review of gas-chromatographic measurement methodologies for atmospheric halogenated greenhouse gases. Critical reviews in analytical chemistry, 1–14. Advance online publication.

[bb0210] Hindle F., Kuuliala L., Mouelhi M., Cuisset A., Bray C., Vanwolleghem M., Devlieghere F., Mouret G., Bocquet R. (2018). Monitoring of food spoilage by high resolution THz analysis. The Analyst.

[bb0215] Hong G.-H., Le T.-C., Lin G.-Y., Cheng H.-W., Yu J.-Y., Dejchanchaiwong R., Tekasakul P., Tsai C.-J. (2023). Long-term field calibration of low-cost metal oxide VOC sensor: Meteorological and interference gas effects. Atmospheric Environment.

[bb0220] Huang Y., Doh I.J., Bae E. (2021). Design and validation of a portable machine learning-based electronic Nose. Sensors (Basel).

[bb0225] Ifmalinda A. (2022). Assessment the volatile organic compound of avocado during ripening process and mechanical damage using electronic-nose system. IOP Conference Series : Earth and Environmental Science.

[bb0230] Ioannidis A.G., Kerckhof F.M., Riahi Drif Y., Vanderroost M., Boon N., Ragaert P., Devlieghere F. (2018). Characterization of spoilage markers in modified atmosphere packaged iceberg lettuce. International Journal of Food Microbiology.

[bb0240] Ji J., Huang H., Li L., Ye J., Sun J., Sheng L., Ye Y., Zheng Y., Zhang Z., Sun X. (2022). Volatile metabolite profiling of wheat kernels contaminated by *fusarium graminearum*. Journal of Agricultural and Food.

[bb0245] Jiang L., Dumlao M.C., Donald W.A., Steel C.C., Schmidtke L.M. (2023). Rapid in-field volatile sampling for detection of *Botrytis cinerea* infection in wine grapes. Molecules (Basel, Switzerland).

[bb0250] Jiarpinijnun A., Osako K., Siripatrawan U. (2020). Visualization of volatomic profiles for early detection of fungal infection on storage jasmine brown rice using electronic nose coupled with chemometrics. Measurement.

[bb0255] Kataoka H., Lord H.L., Pawliszyn J. (2000). Applications of solid-phase microextraction in food analysis. Journal of Chromatography. A.

[bb0260] Ketola R.A., Kotiaho T., Cisper M.E., Allen T.M. (2002). Environmental applications of membrane introduction mass spectrometry. Journal of mass spectrometry : JMS.

[bb0265] Kim C., Lee K.K., Kang M.S., Shin D.M., Oh J.W., Lee C.S., Han D.W. (2022). Artificial olfactory sensor technology that mimics the olfactory mechanism: A comprehensive review. Biomaterials Research.

[bb0270] Kim S.M., Lee S.M., Seo J.-A., Kim Y.-S. (2018). Changes in volatile compounds emitted by fungal pathogen spoilage of apples during decay. Postharvest Biology and Technology.

[bb0275] Kovacevic T.K., Major N., Sivec M., Horvat D., Krpan M., Hruskar M., Goreta Ban S. (2023). Phenolic content, amino acids, volatile compounds, antioxidant capacity, and their relationship in wild garlic (A. Ursinum L.). Foods.

[bb0280] Kuchikata H., Sano M., Fujiwara F., Murashima K., Kumaishi K., Narukawa M., Kusano M. (2024). Soil volatilomics uncovers tight linkage between soybean presence and soil omics profiles in agricultural fields. Scientific Reports.

[bb0285] Kwaśny M., Bombalska A. (2023). Optical methods of methane detection. Sensors (Basel, Switzerland).

[bb0290] Kwon H., Park J., Jang H.W., Lim H., Kim S., Kim S., Choi J.W. (2025). Synergistic integration of laser oxidation and long short-term memory for advanced odor classification in next-generation artificial olfactory systems. ACS Sensors.

[bb0295] Leary P.E., Kizzire K.L., Chan Chao R., Niedziejko M., Martineau N., Kammrath B.W. (2023). Evaluation of portable gas chromatography-mass spectrometry (GC-MS) for the analysis of fentanyl, fentanyl analogs, and other synthetic opioids. Journal of Forensic Sciences.

[bb0300] Lee G.H.O., Jamalzadegan S., Liu Y.X., Wang H.Y., Saville A.C., Shymanovich T., Wei Q.S. (2023). Abaxial leaf surface-mounted multimodal wearable sensor for continuous plant physiology monitoring. Science Advances.

[bb0305] Lew T.T.S., Koman V.B., Silmore K.S., Seo J.S., Gordiichuk P., Kwak S.Y., Strano M.S. (2020). Real-time detection of wound-induced H(2)O(2) signalling waves in plants with optical nanosensors. Nature Plants.

[bb0310] Li G., Wang Y., Zhao Q., Yuan P., Chang B. (2023). PMVT: A lightweight vision transformer for plant disease identification on mobile devices. Frontiers in Plant Science.

[bb0315] Li H., Kang X., Wang S., Mo H., Xu D., Zhou W., Hu L. (2021). Early detection and monitoring for *aspergillus flavus* contamination in maize kernels. Food Control.

[bb0320] Li M., Cappellin L., Xu J., Biasioli F., Varotto C. (2020). High-throughput screening for in planta characterization of VOC biosynthetic genes by PTR-ToF-MS. Journal of Plant Research.

[bb0325] Li M., Dong S., Cao S., Cui Q., Chen Q., Ning J., Li L. (2023). A rapid aroma quantification method: Colorimetric sensor-coupled multidimensional spectroscopy applied to black tea aroma. Talanta.

[bb0330] Li X., Li T., Li M., Chen D., Liu X., Zhao S., Dai X., Chen J., Kong Z., Tan J. (2022). Effect of pathogenic fungal infestation on the berry quality and volatile organic compounds of cabernet sauvignon and petit manseng grapes. Frontiers in Plant Science.

[bb0335] Li Z., Paul R., Ba Tis T., Saville A.C., Hansel J.C., Yu T., Wei Q. (2019). Non-invasive plant disease diagnostics enabled by smartphone-based fingerprinting of leaf volatiles. Nature Plants.

[bb0340] Lin H., Kang W., Kutsanedzie F.Y.H., Chen Q. (2019). A Novel Nanoscaled Chemo Dye–Based Sensor for the Identification of Volatile Organic Compounds During the Mildewing Process of Stored Wheat. Food Analytical Methods.

[bb0345] Lin H., Wang F., Lin J., Yang W., Kang W., Jiang H., Chen Q. (2023). Detection of wheat toxigenic aspergillus flavus based on nano-composite colorimetric sensing technology. Food Chemistry.

[bb0350] Liu Q., Zhao N., Zhou D., Sun Y., Sun K., Pan L., Tu K. (2018). Discrimination and growth tracking of fungi contamination in peaches using electronic nose. Food Chemistry.

[bb0355] Liu X., Huo D., Li J., Ma Y., Liu H., Luo H., Zhang S., Luo X., Hou C. (2023). Pattern-recognizing-assisted detection of mildewed wheat by dyes/dyes-cu-MOF paper-based colorimetric sensor array. Food Chemistry.

[bb0365] Lutz É., Coradi P.C. (2022). Applications of new technologies for monitoring and predicting grains quality stored: Sensors, internet of things, and artificial intelligence. Measurement.

[bb0370] Ma J., Guan Y., Xing F., Eltzov E., Wang Y., Li X., Tai B. (2023). Accurate and non-destructive monitoring of mold contamination in foodstuffs based on whole-cell biosensor array coupling with machine-learning prediction models. Journal of Hazardous Materials.

[bb0375] Ma J., Veltman B., Tietel Z., Tsror L., Liu Y., Eltzov E. (2020). Monitoring of infection volatile markers using CMOS-based luminescent bioreporters. Talanta.

[bb0380] Maffei M.E., Gertsch J., Appendino G. (2011). Plant volatiles: Production, function and pharmacology. Natural Product Reports.

[bb0385] Malik M., Demetrowitsch T., Schwarz K., Kunze T. (2024). New perspectives on 'Breathomics': Metabolomic profiling of non-volatile organic compounds in exhaled breath using DI-FT-ICR-MS. Communications Biology.

[bb0390] Mazzucotelli M., Farneti B., Khomenko I., Gonzalez-Estanol K., Pedrotti M., Fragasso M., Capozzi V., Biasioli F. (2022). Proton transfer reaction mass spectrometry: A green alternative for food volatilome profiling. Green Analytical Chemistry.

[bb0395] Moon Y.K., Kim K.B., Jeong S.Y., Lee J.H. (2022). Designing oxide chemiresistors for detecting volatile aromatic compounds: Recent progresses and future perspectives. Chemical Communications (Cambridge, England).

[bb0405] Moshayedi A.J., Sohail Khan A., Hu J., Nawaz A., Zhu J. (2023). E-Nose-driven advancements in Ammonia gas detection: A comprehensive review from traditional to cutting-edge Systems in Indoor to outdoor agriculture. Sustainability.

[bb0410] Murali-Baskaran R.K., Mooventhan P., Das D., Dixit A., Sharma K.C., Senthil-Nathan S., Ghosh P.K. (2022). The future of plant volatile organic compounds (pVOCs) research: Advances and applications for sustainable agriculture. Environmental and Experimental Botany.

[bb0415] Nagalingam S., Seco R., Kim S., Guenther A. (2023). Heat stress strongly induces monoterpene emissions in some plants with specialized terpenoid storage structures. Agricultural and Forest Meteorology.

[bb0420] Natarajan S., Chakrabarti P., Margala M. (2024). Robust diagnosis and meta visualizations of plant diseases through deep neural architecture with explainable AI. Scientific Reports.

[bb0425] Nath B., Chen G., O’Sullivan C.M., Zare D. (2024). Research and technologies to reduce grain postharvest losses. A Review. Foods.

[bb0430] Navarro E., Costa N., Pereira A. (2020). A systematic review of IoT solutions for smart farming. Sensors (Basel).

[bb0435] Niklas C., Wackerbarth H., Ctistis G. (2021). A short review of cavity-enhanced Raman spectroscopy for gas analysis. Sensors (Basel, Switzerland).

[bb0445] Nouri B., Mohtasebi S.S., Rafiee S. (2020). Quality detection of pomegranate fruit infected with fungal disease. International Journal of Food Properties.

[bb0455] Picazo-Aragones J., Terrab A., Balao F. (2020). Plant volatile organic compounds evolution: Transcriptional regulation, epigenetics and polyploidy. International Journal of Molecular Sciences.

[bb0460] Qaddos A., Yaseen M.U., Al-Shamayleh A.S., Imran M., Akhunzada A., Alharthi S.Z. (2024). A novel intrusion detection framework for optimizing IoT security. Scientific Reports.

[bb0465] Rezende G.C., Le Calvé S., Brandner J.J., Newport D. (2019). Micro milled microfluidic photoionization detector for volatile organic compounds. Micromachines.

[bb0470] Richards L.C., Davey N.G., Fyles T.M., Gill C.G., Krogh E.T. (2018). Discrimination of constructed air samples using multivariate analysis of full scan membrane introduction mass spectrometry (MIMS) data. Rapid communications in mass spectrometry : RCM.

[bb0475] Sanjeevi P., Siva Kumar B., Prasanna S., Maruthupandi J., Manikandan R., Baseera A. (2020). An ontology enabled internet of things framework in intelligent agriculture for preventing post-harvest losses. Complex & Intelligent Systems.

[bb0480] Schuhfried E., Betta E., Cappellin L., Aprea E., Gasperi F., Märk T.D., Biasioli F. (2017). Withering of plucked Trachelospermum jasminoides (star jasmine) flowers – Time-dependent volatile compound profile obtained with SPME/GC–MS and proton transfer reaction-mass spectrometry (PTR-MS). Postharvest Biology and Technology.

[bb0485] Shi H., Zhang M., Adhikari B. (2017). Advances of electronic nose and its application in fresh foods: A review. Critical Reviews in Food Science and Nutrition.

[bb0490] da Silva V., Ferreira M., Barbosa J.L., Kamruzzaman M., Barbin D.F. (2023). Low-cost electronic-nose (LC-e-nose) systems for the evaluation of plantation and fruit crops: Recent advances and future trends. Analytical methods : advancing methods and applications.

[bb0495] Snyder A.B., Martin N., Wiedmann M. (2024). Microbial food spoilage: Impact, causative agents and control strategies. Nature Reviews. Microbiology.

[bb0500] Song C., Hong X., Zhao S., Liu J., Schulenburg K., Huang F.C., Schwab W. (2016). Glucosylation of 4-Hydroxy-2,5-Dimethyl-3(2H)-Furanone, the key strawberry flavor compound in strawberry fruit. Plant Physiology.

[bb0505] Stathers T., Holcroft D., Kitinoja L., Mvumi B.M., English A., Omotilewa O., Torero M. (2020). A scoping review of interventions for crop postharvest loss reduction in sub-Saharan Africa and South Asia. Nature Sustainability.

[bb0510] Sun P., Schuurink R.C., Caissard J.C., Hugueney P., Baudino S. (2016). My way: Noncanonical biosynthesis pathways for plant volatiles. Trends in Plant Science.

[bb0515] Tabassum S., Kumar R., Dong L. (2017). Nanopatterned optical Fiber tip for guided mode resonance and application to gas sensing. IEEE Sensors Journal.

[bb0520] Tantiwanichapan K., Jolivot R., Jomphoak A., Srisuai N., Chananonnawathorn C., Lertvanithpol T., Horprathum M., Boonruang S. (2023). Demonstration of cross reaction in hybrid graphene oxide/tantalum dioxide guided mode resonance sensor for selective volatile organic compound. Scientific Reports.

[bb0525] Tiwari S., Kate A., Mohapatra D., Tripathi M.K., Ray H., Akuli A., Modhera B. (2020). Volatile organic compounds (VOCs): Biomarkers for quality management of horticultural commodities during storage through e-sensing. Trends in Food Science & Technology.

[bb0530] Ulrich D., Kecke S., Olbricht K. (2018). What do we know about the chemistry of strawberry aroma?. Journal of Agricultural and Food Chemistry.

[bb0535] Vandendriessche T., Keulemans J., Geeraerd A., Nicolai B.M., Hertog M.L. (2012). Evaluation of fast volatile analysis for detection of Botrytis cinerea infections in strawberry. Food Microbiology.

[bb0540] Veltman B., Harpaz D., Melamed S., Tietel Z., Tsror L., Eltzov E. (2022). Whole-cell bacterial biosensor for volatile detection from *Pectobacterium*-infected potatoes enables early identification of potato tuber soft rot disease. Talanta.

[bb0545] Voss H.G.J., Stevan S.L., Ayub R.A. (2019). Peach growth cycle monitoring using an electronic nose. Computers and Electronics in Agriculture.

[bb0550] Walters K.J., Lopez R.G., Behe B.K. (2020). Leveraging controlled-environment agriculture to increase key basil Terpenoid and Phenylpropanoid concentrations: The effects of radiation intensity and CO(2) concentration on consumer preference. Frontiers in Plant Science.

[bb0555] Wang J., Jiang H., Chen Q. (2021). High-precision recognition of wheat mildew degree based on colorimetric sensor technique combined with multivariate analysis. Microchemical Journal.

[bb0560] Wang S., Mo H., Xu D., Hu H., Hu L., Shuai L., Li H. (2021). Determination of volatile organic compounds by HS-GC-IMS to detect different stages of *aspergillus flavus* infection in Xiang Ling walnut. Food Science & Nutrition.

[bb0565] Wang X., Bouzembrak Y., Lansink A.O., van der Fels-Klerx H.J. (2022). Application of machine learning to the monitoring and prediction of food safety: A review. Comprehensive Reviews in Food Science and Food Safety.

[bb0570] Wang X., Liu J. (2024). Vegetable disease detection using an improved YOLOv8 algorithm in the greenhouse plant environment. Scientific Reports.

[bb0575] Weraduwage S.M., Rasulov B., Sahu A., Niinemets U., Sharkey T.D. (2022). Isoprene measurements to assess plant hydrocarbon emissions and the methylerythritol pathway. Methods in Enzymology.

[bb0580] Wu J., Cao J., Chen J., Huang L., Wang Y., Sun C., Sun C. (2023). Detection and classification of volatile compounds emitted by three fungi-infected citrus fruit using gas chromatography-mass spectrometry. Food Chemistry.

[bb0585] Wu Q., Yuan Y., Wang X., Bu X., Jiao M., Liu W., Han C., Hu L., Wang X., Li X. (2023). Highly selective ionic gel-based gas sensor for halogenated volatile organic compound detection: Effect of dipole-dipole interaction. ACS Sensors.

[bb0590] Yang S., Meng Z., Li Y., Chen R., Yang Y., Zhao Z. (2021). Evaluation of physiological characteristics, soluble sugars, organic acids and volatile compounds in 'Orin' apples (Malus domestica) at different ripening stages. Molecules.

[bb0595] You D.W., Seon Y.S., Jang Y., Bang J., Oh J.S., Jung K.W. (2020). A portable gas chromatograph for real-time monitoring of aromatic volatile organic compounds in air samples. Journal of Chromatography. A.

[bb0600] Zhang J., Zhang B., Dong J., Tian Y., Lin Y., Fang G., Wang S. (2022). Identification of mouldy rice using an electronic nose combined with SPME-GC/MS. Journal of Stored Products Research.

[bb0605] Zhang W., Chen W., Pan H., Sanaeifar A., Hu Y., Shi W., Guo J., Ding L., Zhou J., Li X., He Y. (2024). Rapid identification of the aging time of Liupao tea using AI-multimodal fusion sensing technology combined with analysis of tea polysaccharide conjugates. International Journal of Biological Macromolecules.

[bb0610] Zhang Y., Zhu D., Ren X., Shen Y., Cao X., Liu H., Li J. (2022). Quality changes and shelf-life prediction model of postharvest apples using partial least squares and artificial neural network analysis. Food Chemistry.

[bb0615] Zhou J., Al Husseini D., Li J., Lin Z., Sukhishvili S., Cote G.L., Lin P.T. (2022). Detection of volatile organic compounds using mid-infrared silicon nitride waveguide sensors. Scientific Reports.

[bb0620] Zhu J., Ji S., Ren Z., Wu W., Zhang Z., Ni Z., Liu L., Zhang Z., Song A., Lee C. (2023). Triboelectric-induced ion mobility for artificial intelligence-enhanced mid-infrared gas spectroscopy. Nature Communications.

[bb0625] Zhu J., Wen H., Fan Y., Yang X., Zhang H., Wu W., Zhou Y., Hu H. (2022). Recent advances in gas and environmental sensing: From micro/nano to the era of self-powered and artificial intelligent (AI)-enabled device. Microchemical Journal.

[bb0630] Zytek A., Rusinek R., Oniszczuk A., Gancarz M. (2023). Effect of the consolidation level on organic volatile compound emissions from maize during storage. Materials (Basel).

