# Supplementary material for: A comprehensive review of post-harvest agricultural product deterioration signature volatile organic compounds
Source: Food Chem X. 2025 Aug 4;29:102866. doi: 10.1016/j.fochx.2025.102866 (PMC12345322; doi:10.1016/j.fochx.2025.102866)
Supplement: Supplementary file 1 — Supplementary material [file mmc1.docx]

**Supplementary Material**


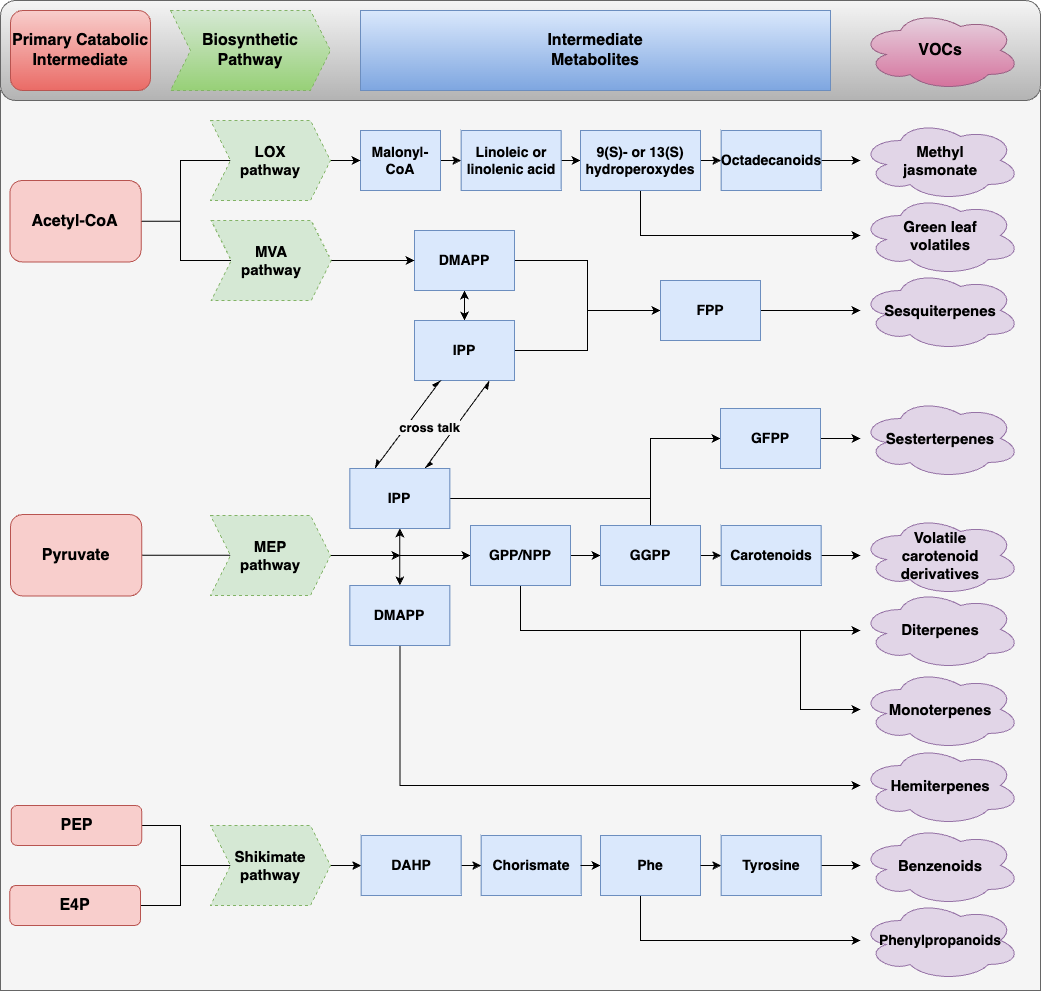


Figure S1. Overview of biosynthetic pathways leading to the emission of plant VOCs(Dudareva et al., 2013).


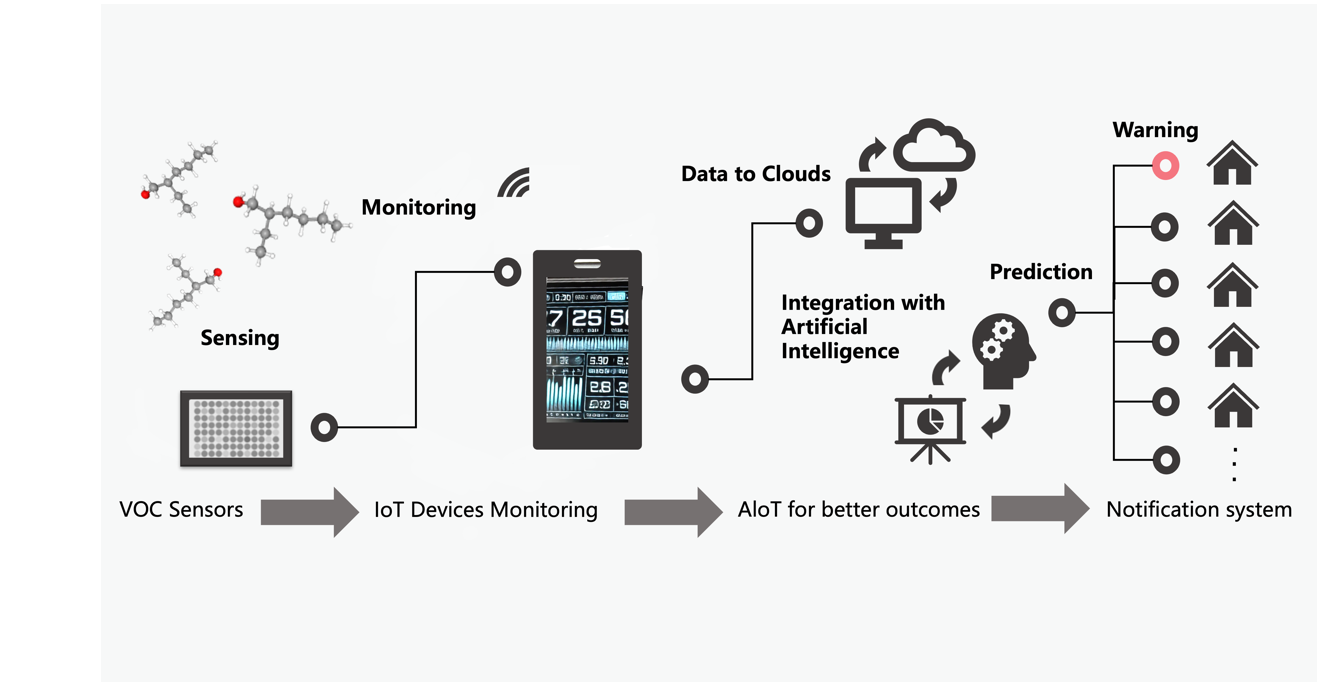


Figure S2. From smart "Things" in IoT systems to adopting Artificial Intelligence techniques in deterioration prediction
